# Maternal Nativity, Race, and Ethnicity and Infant Mortality in the US

**DOI:** 10.1001/jamanetworkopen.2025.52230

**Published:** 2026-01-06

**Authors:** Nicolette Christodoulakis, Giulia M. Muraca

**Affiliations:** 1 Department of Obstetrics and Gynecology, Faculty of Health Sciences, McMaster University, Hamilton, Ontario, Canada; 2 Department of Health Research Methods, Evidence, and Impact, Faculty of Health Sciences, McMaster University, Hamilton, Ontario, Canada; 3 Department of Medicine, Solna, Karolinska Institutet, Eugeniahemmet, Clinical Epidemiology Unit, Stockholm, Sweden

## Abstract

**Importance:**

Racial disparities in infant mortality in the US are well-established, but the associations among maternal nativity status, race, ethnicity, and infant mortality remain understudied.

**Objective:**

To examine the associations among maternal nativity status and infant mortality by maternal race and ethnicity in the US, and to assess whether these associations differ by gestational age.

**Design, Setting, and Participants:**

This retrospective, population-based cohort study used linked birth and infant death records for all recorded US live births from 2016 to 2022. Births to foreign residents and those with missing data on maternal nativity, race, ethnicity, or gestational age were excluded.

**Exposure:**

Maternal nativity status (US born vs non–US born).

**Main Outcomes and Measures:**

The primary outcome was infant mortality, defined as death within 364 days of life. Logistic regression was used to quantify the association between maternal nativity status and infant mortality, adjusting for maternal and infant characteristics. Leading causes of death among preterm (<37 weeks) and full-term (≥37 weeks) births were examined by nativity. Full-term births were further analyzed by race and ethnicity.

**Results:**

Among 25 981 364 births, 20 141 084 (77.5%) were to US-born individuals, and 5 840 280 (22.5%) were to non–US-born individuals (mean [SD] age, 29.1 [5.8] years). US-born individuals had a higher infant mortality rate than non–US-born individuals (5.4 vs 4.0 deaths per 1000 births; adjusted odds ratio [aOR], 1.34; 95% CI, 1.32-1.36). This disparity was primarily seen for full-term births (aOR, 1.58; 95% CI, 1.54-1.63) and was less apparent among preterm births. Among full-term births, higher rates of infant mortality in US-born individuals were found among those identifying as Black (aOR, 1.55; 95% CI, 1.45-1.66), Hispanic (aOR, 1.39; 95% CI, 1.32-1.45), White (aOR, 1.45; 95% CI, 1.35-1.55), and more than 1 race (aOR, 1.33; 95% CI, 1.04-1.69). Leading causes of death differed by nativity and race and ethnicity, with US-born individuals having higher odds of sudden unexpected infant death compared with their non–US-born counterparts among full-term births (aOR, 2.90; 95% CI, 2.74-3.07).

**Conclusions and Relevance:**

This population-based cohort study found that US-born individuals had significantly higher odds of infant mortality compared with non–US-born individuals, particularly among full-term births and among those self-identifying as Black, Hispanic, White, or more than 1 race. Sudden unexpected infant death was a major contributor to these disparities. Investigation into the underlying factors contributing to these disparities is needed.

## Key Points

**Question:**

In the US, is maternal nativity or race and ethnicity associated with infant mortality, and do these associations vary by gestational age?

**Findings:**

In this cohort study of more than 25 million births from 2016 to 2022, infant mortality rates were higher among US-born birthing individuals compared with non–US-born birthing individuals. This disparity was accounted for by full-term births among those who were Black, Hispanic, White and multiracial.

**Meaning:**

These findings suggest that maternal nativity status is associated with infant mortality across racial and ethnic groups, highlighting the need for investigation into the underlying factors contributing to these disparities, particularly among full-term births.

## Teaser

This cohort study examines the associations among the birthing parent’s nativity status and race and infant mortality in the US and assesses whether these associations differ by gestational age.

## Introduction

Although racial and ethnic disparities in infant mortality in the US are well documented^1^ and have remained largely unchanged over the last decade,^1,2,3,4,5,6,7^ the intersection between race, ethnicity, and nativity status, defined by whether the birthing person was born in or outside the US, remains understudied. One of the earliest studies to examine this association in 1983 to 1984 found that Black mothers who were born outside the US had 28% lower infant mortality rates than their US-born counterparts, with no difference observed among White mothers.^8^ Since then, multiple studies have continued to investigate infant outcomes by nativity, noting a recurring phenomenon often referred to as the immigrant birth paradox.^9^ This is the observation that non–US-born mothers, despite facing socioeconomic disadvantages and systemic barriers to health care access, often have better perinatal outcomes than their US-born racial and ethnic counterparts. However, this paradox is not uniform across all groups, and variability exists both within and between racial subpopulations.

A recent review summarized the existing literature on US perinatal outcomes by the nativity status of the birthing person and found that although most studies focus on low birth weight and preterm birth, fewer examine infant mortality.^10^ Those that do study this association consistently document lower infant mortality rates among foreign-born mothers.^10^ These findings have been observed across a range of contexts, including Mexican communities in Los Angeles,^11^ Black and Filipino communities in California,^12,13^ as well as Mexican, Cuban, other Hispanic, non-Hispanic Black, and non-Hispanic White individuals in the US.^14^

Despite these findings, critical gaps in the literature remain. Many studies that have included multiple racial groups are outdated, and their conclusions may no longer reflect current demographic patterns or the evolving health care landscape. More recent literature also tends to focus on a specific race, reducing the generalizability of the findings. Furthermore, most studies do not differentiate between full-term and preterm births in analyses of infant mortality, often combining all births or focusing on just 1 subgroup, potentially masking important differences. To address these gaps, we aimed to examine the association between maternal nativity and infant mortality by maternal race and ethnicity and gestational age. As a secondary objective, we investigated differences in causes of infant death by nativity status and race and ethnicity.

## Methods

### Study Design and Population

We conducted a population-based, retrospective cohort study of all live births in the US between 2016 and 2022, excluding births to individuals who reside outside the US. Data were obtained from the National Vital Statistics System Period/Cohort Linked Birth-Infant Death Data Files. These files include nearly 100% of all linked birth and infant death records reported to the National Center for Health Statistics. This study used publicly available, deidentified data and, in accordance with the Tri-Council Policy Statement Article 2.2, did not require research ethics review or informed consent.^15^ This study adhered to the Strengthening the Reporting of Observational Studies in Epidemiology (STROBE) reporting guidelines for cohort studies.

### Primary Exposure

Our primary exposure was maternal nativity status, identified through the 2003 revision of the US Standard Certificate of Live Birth, which asks mothers to report their place of birth.^16^ Birthing individuals born in the US were classified as US born, whereas those born outside the US were classified as non–US born.

Maternal race was self-identified at time of birth and reported separately from Hispanic origin, following 1997 Office of Management and Budget standards and single-race reporting.^17,18^ For this analysis, we used the National Vital Statistics System natality file categorization and defined each group as follows: non-Hispanic American Indian and Alaska Native (hereafter, American Indian and Alaska Native), non-Hispanic Asian (hereafter, Asian), non-Hispanic Black (hereafter, Black), non-Hispanic Native Hawaiian or Other Pacific Islander (hereafter, Native Hawaiian or Other Pacific Islander), non-Hispanic White (hereafter, White), non-Hispanic individuals reporting more than 1 race (hereafter, more than 1 race), and Hispanic ethnicity.

### Primary Outcome

Our primary outcome was infant mortality, defined as death of a live-born infant before age 1 year. Deaths were identified through linked death certificates in the National Vital Statistics System files. Cause of death was classified using the *International Statistical Classification of Diseases and Related Health Problems, Tenth Revision (ICD-10)*.^19^

### Statistical Analysis

Demographic and clinical characteristics were compared by nativity for both preterm and full-term births. Infant mortality rates were calculated as the number of deaths per 1000 live births. We examined unadjusted infant mortality rates by maternal nativity status across the full cohort and stratified our analyses by maternal race and by gestational age (preterm, <37 weeks; full-term, ≥37 weeks).

We used logistic regression to examine the association between maternal nativity and infant mortality, overall and stratified by maternal race and ethnicity using adjusted odds ratios (aORs) with 95% CIs. Statistical significance was defined as a 95% CI that did not contain the null value of 1. Grouped classifications of cause of death were defined using the 130 selected causes of infant death from the *ICD-10* (eTable 1 in Supplement 1). Additional models were fit in cohorts restricted to preterm and full-term births. All regression models were adjusted for the following maternal characteristics: age, education, marital status, insurance status, cigarette use (before or during pregnancy), and prenatal care. To account for differences in infant characteristics between the US-born and non–US-born groups, we created a second model adjusting for all the maternal characteristics from the first model as well as birth weight, severe neonatal morbidity (defined as seizure, 5-minute Apgar score <4, and assisted ventilation >6 hours), and neonatal intensive care unit admission. A complete-case analysis was performed where observations with missing covariate data were excluded if missingness was less than 3%. If missingness for a covariate exceeded 3%, it was included as a missing category in the adjusted models.

To investigate causes of infant death, we focused on those accounting for at least 3% of infant deaths during the study period, analyzed separately for preterm and full-term births. Cause-specific infant mortality rates were calculated as the number of deaths per 100 000 births and stratified by maternal nativity. We further analyzed cause-specific mortality only for racial and ethnic groups in which significant differences in infant mortality were observed by maternal nativity. Cause of death was classified using *ICD-10* codes (eTable 1 in Supplement 1).

In sensitivity analyses, we constructed 2 additional logistic regression models to examine associations between maternal nativity and the 2 leading causes of infant death among full-term births: (1) sudden unexpected infant death (SUID; defined as a composite of accidental suffocation and strangulation in bed; other symptoms, signs, and abnormal clinical and laboratory findings not elsewhere classified [unspecified causes]; and sudden infant death syndrome [SIDS]) and (2) congenital malformations, deformations, and chromosomal abnormalities. All statistical analyses were performed using SAS statistical software version 9.4 (SAS Institute Inc).

## Results

The study included 25 981 364 live births, of which 20 141 084 (77.5%) were to US-born birthing individuals and 5 840 280 (22.5%) were to non–US-born birthing individuals (mean [SD] age, 29.1 [5.8] years). Among all births, 2 628 504 (10.1%) were preterm and 23 352 860 (89.9%) were full term. In the total population, 196 943 individuals (0.8%) self-identified as American Indian and Alaska Native, 1 629 571 (6.3%) as Asian, 3 762 573 (14.5%) as Black, 6 268 587 (24.1%) as Hispanic, 66 201 (0.3%) as Native Hawaiian or Other Pacific Islander, 13 468 032 (51.8%) as White, and 589 457 (2.3%) as more than 1 race. Among US-born individuals, there were higher proportions of individuals of White race, younger maternal age, postsecondary education, and private insurance, whereas among non–US-born individuals, there were greater proportions of individuals of Hispanic ethnicity, older maternal age, less than high school education, and Medicaid insurance status (Table).

**Table.** Characteristics of Preterm and Full-Term Births in the US by Nativity, 2016-2022

Characteristics	Births, No. (%)					
	Preterm			Full term		
	US-born mother	Non–US-born mother	Total	US-born mother	Non–US-born mother	Total
All births	2 099 390 (79.9)	529 114 (20.1)	2 628 504 (100.0)	18 041 694 (77.3)	5 311 166 (22.7)	23 352 860 (100.0)
Year of birth						
2016	304 177 (79.4)	79 095 (20.6)	383 272 (14.6)	2 694 984 (76.6)	821 369 (23.4)	3 516 353 (15.1)
2017	300 717 (79.6)	77 295 (20.4)	378 012 (14.4)	2 634 061 (76.7)	802 223 (23.3)	3 436 284 (14.7)
2018	298 991 (79.7)	76 183 (20.3)	375 174 (14.3)	2 599 302 (77.0)	775 209 (23.0)	3 374 511 (14.5)
2019	302 091 (79.9)	75 946 (20.1)	378 037 (14.4)	2 567 686 (77.2)	757 736 (22.8)	3 325 422 (14.2)
2020	289 531 (80.5)	70 316 (19.5)	359 847 (13.7)	2 501 403 (77.9)	710 425 (22.1)	3 211 828 (13.8)
2021	305 489 (80.7)	73 142 (19.3)	378 631 (14.4)	2 542 762 (78.5)	697 345 (21.5)	3 240 107 (13.9)
2022	298 394 (79.5)	77 137 (20.5)	375 531 (14.3)	2 501 496 (77.0)	746 859 (23.0)	3 248 355 (13.9)
Maternal age, y						
≤19	111 094 (88.2)	14 908 (11.8)	126 002 (4.8)	937 768 (86.8)	142 697 (13.2)	1 080 465 (4.6)
20-24	418 337 (87.6)	59 085 (12.4)	477 422 (18.2)	3 742 520 (84.5)	687 976 (15.5)	4 430 496 (19.0)
25-34	1 167 786 (80.8)	277 608 (19.2)	1 445 394 (55.0)	10 495 486 (77.3)	3 090 520 (22.7)	13 586 006 (58.2)
35-44	395 139 (69.8)	171 014 (30.2)	566 153 (21.5)	2 839 007 (67.5)	1 367 359 (32.5)	4 206 366 (18.0)
≥45	7034 (52.0)	6499 (48.0)	13 533 (0.5)	26 913 (54.3)	22 614 (45.7)	49 527 (0.2)
Maternal race and ethnicity						
American Indian and Alaska Native	23 001 (99.0)	233 (1.0)	23 234 (0.9)	171 524 (98.7)	2185 (1.3)	173 709 (0.7)
Asian	32 068 (22.5)	110 545 (77.5)	142 613 (5.4)	298 534 (20.1)	1 188 424 (79.9)	1 486 958 (6.4)
Black	471 214 (87.8)	65 219 (12.2)	536 433 (20.4)	2 659 589 (82.4)	566 551 (17.6)	3 226 140 (13.8)
Hispanic	340 794 (55.3)	275 941 (44.7)	616 735 (23.5)	3 014 326 (53.3)	2 637 526 (46.7)	5 651 852 (24.2)
Native Hawaiian and Other Pacific Islander	2628 (34.1)	5078 (65.9)	7706 (0.3)	21 603 (36.9)	36 892 (63.1)	58 495 (0.3)
White	1 172 094 (94.6)	67 219 (5.4)	1 239 313 (47.1)	11 399 588 (93.2)	829 131 (6.8)	12 228 719 (52.4)
>1 Race	57 591 (92.2)	4879 (7.8)	62 470 (2.4)	476 530 (90.4)	50 457 (9.6)	526 987 (2.3)
Maternal education						
Less than high school	232 990 (64.4)	129 030 (35.6)	362 020 (13.8)	1 607 029 (57.1)	1 205 106 (42.9)	2 812 135 (12.0)
High school or General Educational Development completed	609 688 (82.6)	128 842 (17.4)	738 530 (28.1)	4 689 076 (78.8)	1 261 678 (21.2)	5 950 754 (25.5)
Postsecondary education	1 239 268 (82.6)	260 624 (17.4)	1 499 892 (57.1)	11 640 620 (80.9)	2 750 077 (19.1)	14 390 697 (61.6)
Unknown	17 444 (62.2)	10 618 (37.8)	28 062 (1.1)	104 969 (52.7)	94 305 (47.3)	199 274 (0.9)
Maternal marital status						
Married	997 340 (77.2)	294 167 (22.8)	1 291 507 (49.1)	9 696 018 (76.1)	3 051 008 (23.9)	12 747 026 (54.6)
Unmarried	953 883 (85.6)	161 051 (14.4)	1 114 934 (42.4)	6 831 168 (82.3)	1 465 862 (17.7)	8 297 030 (35.5)
Unknown or not stated	148 167 (66.7)	73 896 (33.3)	222 063 (8.4)	1 514 508 (65.6)	794 296 (34.4)	2 308 804 (9.9)
Women, Infants, and Children program enrollment						
Yes	733 065 (77.1)	217 390 (22.9)	950 455 (36.2)	5 699 914 (72.1)	2 203 640 (27.9)	7 903 554 (33.8)
No	1 342 484 (81.5)	303 867 (18.5)	1 646 351 (62.6)	12 177 565 (80.0)	3 037 208 (20.0)	15 214 773 (65.2)
Unknown or not stated	23 841 (75.2)	7857 (24.8)	31 698 (1.2)	164 215 (70.0)	70 318 (30.0)	234 533 (1.0)
Prepregnancy body mass index^a^						
<18.5	71 301 (80.6)	17 207 (19.4)	88 508 (3.4)	496 373 (72.4)	188 961 (27.6)	685 334 (2.9)
18.5-24.9	723 075 (78.1)	202 440 (21.9)	925 515 (35.2)	7 196 222 (75.5)	2 338 575 (24.5)	9 534 797 (40.8)
25.0-29.9	498 103 (76.4)	154 148 (23.6)	652 251 (24.8)	4 628 507 (75.0)	1 542 220 (25.0)	6 170 727 (26.4)
≥30.0	744 115 (84.8)	133 199 (15.2)	877 314 (33.4)	5 404 609 (83.4)	1 078 445 (16.6)	6 483 054 (27.8)
Unknown or not stated	62 796 (74.0)	22 120 (26.0)	84 916 (3.2)	315 983 (66.0)	162 965 (34.0)	478 948 (2.1)
Delivery payment source						
Medicaid	977 557 (79.3)	255 846 (20.7)	1 233 403 (46.9)	7 114 808 (73.8)	2 519 519 (26.2)	9 634 327 (41.3)
Private insurance	996 295 (83.2)	200 573 (16.8)	1 196 868 (45.5)	9 695 891 (82.4)	2 068 728 (17.6)	11 764 619 (50.4)
Self-pay	39 790 (45.4)	47 788 (54.6)	87 578 (3.3)	462 779 (48.2)	497 120 (51.8)	959 899 (4.1)
Other	71 489 (77.5)	20 721 (22.5)	92 210 (3.5)	652 151 (77.4)	190 524 (22.6)	842 675 (3.6)
Unknown	14 259 (77.3)	4186 (22.7)	18 445 (0.7)	116 065 (76.7)	35 275 (23.3)	151 340 (0.6)
Maternal cigarette use						
Yes	212 395 (98.0)	4356 (2.0)	216 751 (8.3)	1 259 630 (97.9)	27 580 (2.1)	1 287 210 (5.5)
No	1 874 257 (78.2)	522 668 (21.8)	2 396 925 (91.2)	16 710 282 (76.0)	5 267 053 (24.0)	21 977 335 (94.1)
Unknown or not stated	12 738 (85.9)	2090 (14.1)	14 828 (0.6)	71 782 (81.3)	16 533 (18.7)	88 315 (0.4)
Prenatal care						
Yes	1 923 159 (79.8)	485 450 (20.2)	2 408 609 (91.6)	17 432 283 (77.5)	5 070 592 (22.5)	22 502 875 (96.4)
No	94 265 (81.6)	21 263 (18.4)	115 528 (4.4)	242 697 (70.4)	102 016 (29.6)	344 713 (1.5)
Unknown or not stated	81 966 (78.5)	22 401 (21.5)	104 367 (4.0)	366 714 (72.6)	138 558 (27.4)	505 272 (2.2)
Sex of infant						
Female	987 672 (80.1)	244 622 (19.9)	1 232 294 (46.9)	8 856 791 (77.3)	2 606 830 (22.7)	11 463 621 (49.1)
Male	1 111 718 (79.6)	284 492 (20.4)	1 396 210 (53.1)	9 184 903 (77.3)	2 704 336 (22.7)	11 889 239 (50.9)
Plurality						
Singleton	1 668 506 (79.4)	433 590 (20.6)	2 102 096 (80.0)	17 780 365 (77.2)	5 243 590 (22.8)	23 023 955 (98.6)
Twin	413 154 (82.0)	90 555 (18.0)	503 709 (19.2)	261 074 (79.5)	67 486 (20.5)	328 560 (1.4)
Triplet or higher order	17 730 (78.1)	4969 (21.9)	22 699 (0.9)	255 (73.9)	90 (26.1)	345 (<0.1)
Fetal presentation						
Breech	282 726 (80.1)	70 215 (19.9)	352 941 (13.4)	538 320 (77.4)	157 310 (22.6)	695 630 (3.0)
Cephalic	1 771 958 (79.8)	447 809 (20.2)	2 219 767 (84.4)	17 299 958 (77.3)	5 089 693 (22.7)	22 389 651 (95.9)
Other	35 647 (79.9)	8952 (20.1)	44 599 (1.7)	149 637 (75.6)	48 313 (24.4)	197 950 (0.8)
Unknown or not stated	9059 (80.9)	2138 (19.1)	11 197 (0.4)	53 779 (77.2)	15 850 (22.8)	69 629 (0.3)
Birth weight, g						
<1500	285 757 (80.2)	70 470 (19.8)	356 227 (13.6)	3605 (78.0)	1016 (22.0)	4621 (<0.1)
1500-2499	912 903 (80.1)	227 245 (19.9)	1 140 148 (43.4)	520 968 (78.4)	143 776 (21.6)	664 744 (2.8)
2500-3999	884 458 (79.6)	227 338 (20.4)	1 111 796 (42.3)	15 945 158 (76.9)	4 781 013 (23.1)	20 726 171 (88.8)
≥4000	14 262 (79.5)	3673 (20.5)	17 935 (0.7)	1 567 440 (80.3)	384 418 (19.7)	1 951 858 (8.4)
Not stated	2010 (83.8)	388 (16.2)	2398 (0.1)	4523 (82.7)	943 (17.3)	5466 (<0.1)
Severe neonatal morbidity^b^						
Yes	298 662 (83.6)	58 677 (16.4)	357 339 (13.6)	166 450 (82.4)	35 566 (17.6)	202 016 (0.9)
No	1 800 493 (79.3)	470 387 (20.7)	2 270 880 (86.4)	17 873 679 (77.2)	5 275 231 (22.8)	23 148 910 (99.1)
Unknown or not stated	235 (82.5)	50 (17.5)	285 (<0.1)	1565 (80.9)	369 (19.1)	1934 (<0.1)
Admission to neonatal intensive care unit						
Yes	1 062 558 (80.6)	255 474 (19.4)	1 318 032 (50.1)	828 532 (77.4)	241 982 (22.6)	1 070 514 (4.6)
No	1 034 607 (79.1)	273 088 (20.9)	1 307 695 (49.8)	17 196 022 (77.2)	5 064 476 (22.8)	22 260 498 (95.3)
Unknown or not stated	2225 (80.1)	552 (19.9)	2777 (0.1)	17 140 (78.5)	4708 (21.5)	21 848 (0.1)

^a^ Body mass index is calculated as weight in kilograms divided by height in meters squared.

^b^ Severe neonatal morbidity was defined as 1 or more of any of the following complications: neonatal seizures, 5-minute Apgar score less than 4, and assisted ventilation longer than 6 hours.

### Crude Rate of Infant Mortality by Maternal Nativity Status and Race

**Figure 1.**
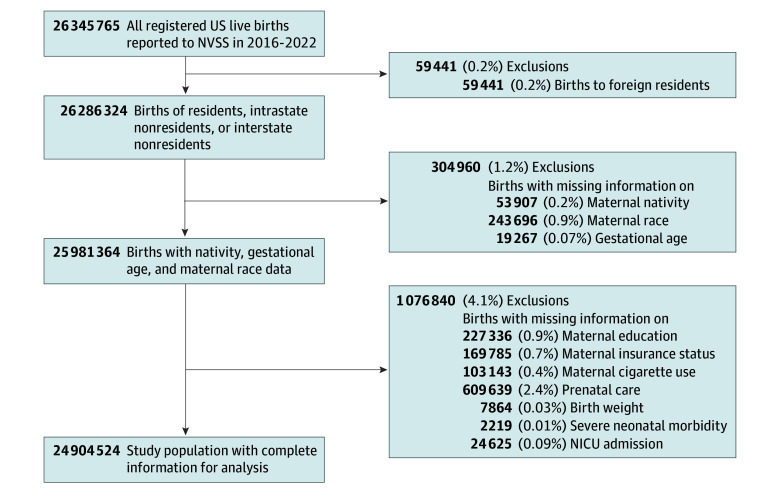
Cohort Enrollment Flowchart NICU indicates neonatal intensive care unit; NVSS, National Vital Statistics System.

For a complete-case analysis, we excluded observations with missing data on maternal education, maternal insurance status, maternal cigarette use, prenatal care, birth weight, severe neonatal morbidity, and neonatal intensive care unit admission (1 076 840 observations [4.1%]) (Figure 1). After these exclusions, 24 904 524 births were included in our final study population for analysis. Among all births, there were 127 264 infant deaths, resulting in an infant mortality rate of 5.1 deaths per 1000 births (5.4 deaths per 1000 births among US-born individuals and 4.0 deaths per 1000 births among non–US-born individuals).

Across all study years, the crude infant mortality rate was consistently higher among US-born compared with non–US-born individuals. A general decline in infant mortality was observed over time, with a decrease among non–US-born individuals in 2020, followed by an increase in 2021 and a subsequent decrease in 2022 (eFigure 1 in Supplement 1). Among US-born individuals, rates remained relatively stable, with a general decline over time and a slight increase between 2021 and 2022. Similar patterns were observed among full-term births, although a more pronounced increase in mortality was seen among US-born individuals between 2020 and 2022. For preterm births, rates declined steadily among US-born individuals but fluctuated among non–US-born individuals. Unlike full-term births, infant mortality rates for preterm births were often similar between nativity groups (eFigure 1 in Supplement 1).

When stratified by maternal race and ethnicity, crude infant mortality rates were higher among US-born vs non–US-born individuals across several racial groups, including Asian (3.6 vs 3.1 deaths per 1000 births), Black (10.5 vs 6.6 deaths per 1000 births), Hispanic (4.8 vs 4.2 deaths per 1000 births), White (4.3 vs 2.9 deaths per 1000 births), and more than 1 race (6.4 vs 4.0 deaths per 1000 births). Differences between US-born and non–US-born individuals were not significant among American Indian and Alaska Native (7.9 vs 4.5 deaths per 1000 births) and Native Hawaiian or Other Pacific Islander (7.1 vs 7.7 deaths per 1000 births) groups. The rate in the Native Hawaiian or Other Pacific Islander group was higher among non–US-born individuals, which diverged from the overall trend observed in the other race groups.

**Figure 2.**
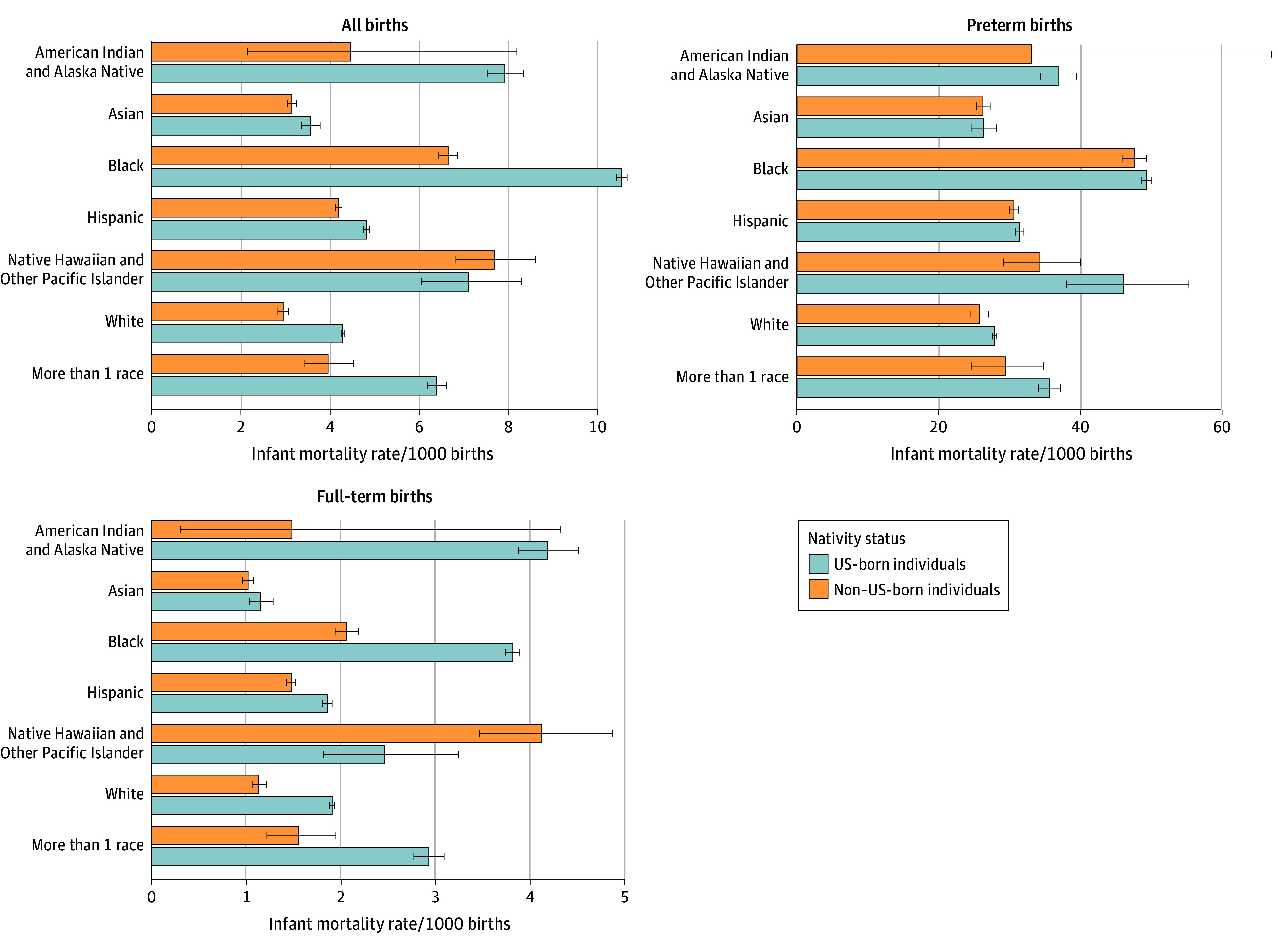
Infant Mortality Rate Among All Births, Preterm Births, and Full-Term Births in the US by Maternal Nativity and Race Error bars denote 95% CIs.

When disaggregated by gestational age, the observed differences by nativity were not apparent among preterm births but were pronounced among full-term births. Among preterm births, the infant mortality rate was 33.1 deaths per 1000 births. The rate among US-born individuals was higher compared with non–US-born individuals (33.6 vs 31.2 deaths per 1000 preterm births); however, differences according to maternal race were minimal. Among full-term births, the overall infant mortality rate was 2.0 deaths per 1000 births and was higher among US-born vs non–US-born individuals (2.2 vs 1.4 deaths per 1000 births). US-born individuals had higher infant mortality rates per 1000 full-term births among individuals who identified as Black (3.8 vs 2.1 deaths per 1000 births), Hispanic (1.8 vs 1.5 deaths per 1000 births), White (1.9 vs 1.1 deaths per 1000 births), and more than 1 race (2.9 vs 1.5 deaths per 1000 births). In the Native Hawaiian or Other Pacific Islander group, the pattern was reversed where US-born individuals had a lower infant mortality rate compared with their non–US-born counterparts (2.4 vs 4.1 deaths per 1000 births) (Figure 2).

### Adjusted Association Between Maternal Nativity and Infant Mortality

Among all births, US-born individuals had significantly higher adjusted odds of infant mortality compared with non–US-born individuals (aOR, 1.34; 95% CI, 1.32-1.36). This pattern remained consistent when stratified by maternal race. Higher adjusted odds of infant mortality were observed among US-born individuals who identified as Asian (aOR, 1.12; 95% CI, 1.05-1.20), Black (aOR, 1.48; 95% CI, 1.42-1.53), Hispanic (aOR, 1.27; 95% CI, 1.24-1.30), White (aOR, 1.30; 95% CI, 1.25-1.35), and more than 1 race (aOR, 1.32; 95% CI, 1.14-1.52). No significant association between nativity and infant mortality was observed in the American Indian and Alaska Native or Native Hawaiian or Other Pacific Islander groups.

Among preterm births, the overall adjusted odds of infant mortality in US-born individuals was reduced compared with the overall population; however, this group continued to have slightly higher odds of infant mortality compared with non–US-born individuals (aOR, 1.03; 95% CI, 1.01-1.05). When stratified by maternal race, there was not a significant association between nativity and infant mortality for most groups. However, among individuals who identified as Native Hawaiian or Other Pacific Islander, US-born individuals had significantly higher odds of infant mortality compared with non–US-born individuals (aOR, 1.32; 95% CI, 1.01-1.73).

**Figure 3.**
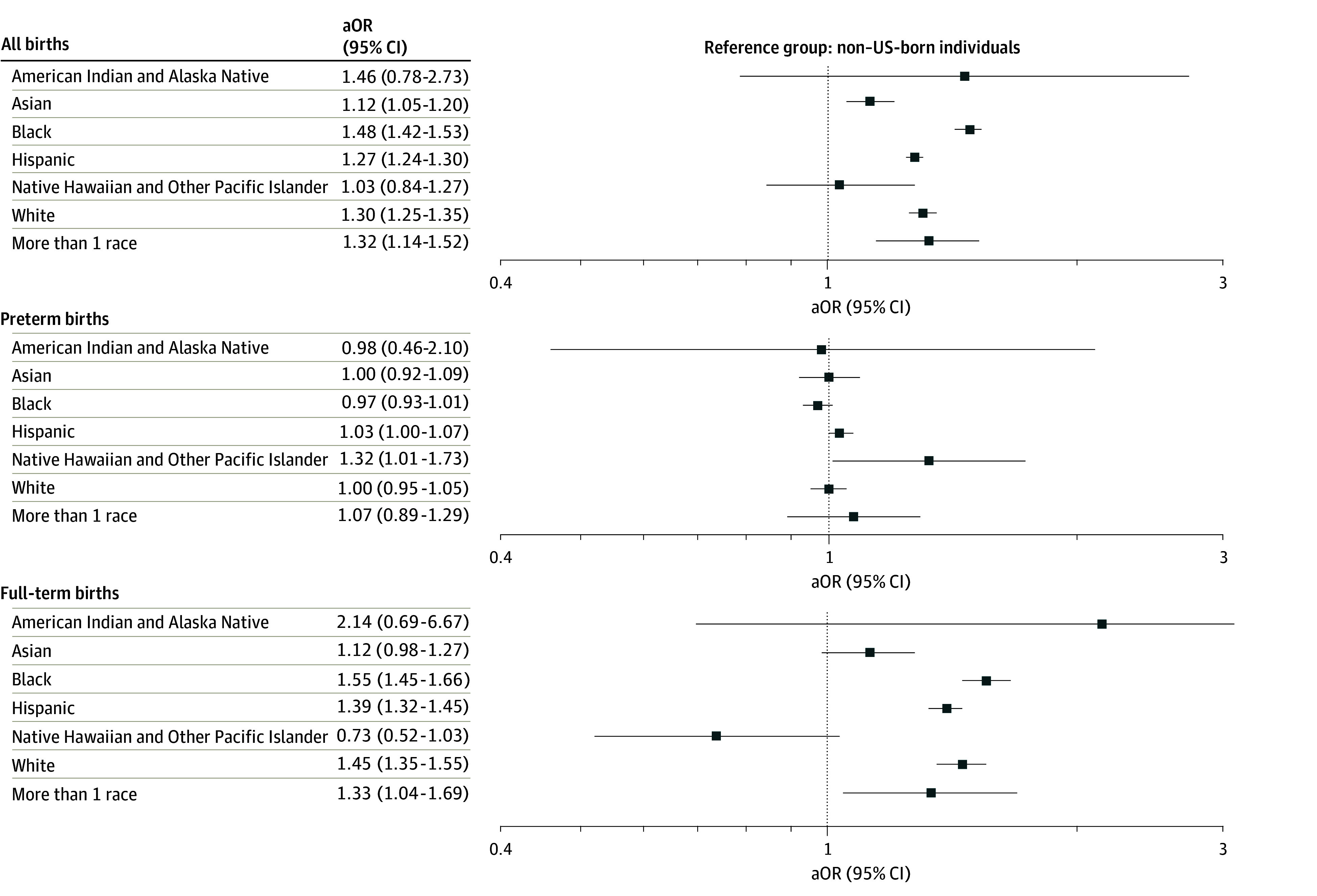
Adjusted Odds Ratios (aORs) and 95% CIs Showing the Association Between Maternal Nativity and Infant Mortality Among All Births, Preterm Births, and Full-Term Births by Maternal Race

Among full-term births, US-born individuals had higher adjusted odds of infant mortality compared with non–US-born individuals (aOR, 1.58; 95% CI, 1.54-1.63). This association was consistent across multiple racial groups, including Black (aOR, 1.55; 95% CI, 1.45-1.66), Hispanic (aOR, 1.39; 95% CI, 1.32-1.45), White (aOR, 1.45; 95% CI, 1.35-1.55), and more than 1 race (aOR, 1.33; 95% CI, 1.04-1.69) (Figure 3). The addition of infant characteristics attenuated the associations seen among Asian individuals in the overall cohort and among Native Hawaiian or Other Pacific Islander individuals in the preterm cohort but did not alter the observed associations among full-term births. Full frequency counts of births and deaths, crude rates of infant mortality, and aORs by maternal nativity and race are presented in eTable 2 in Supplement 1.

### Causes of Death Among Preterm and Full-Term Births

The leading cause of death among preterm births included extremely low birth weight or extreme immaturity (587.9 deaths per 100 000 births), other perinatal conditions (222.4 deaths per 100 000 births), other low birth weight or preterm (169.0 deaths per 100 000 births), newborn affected by premature rupture of membranes (144.6 deaths per 100 000 births), bacterial sepsis of newborn (130.7 deaths per 100 000 births), and respiratory distress of newborn (104.1 deaths per 100 000 births). Among preterm births, the rates for the leading causes of death were comparable by nativity (eFigure 2 in Supplement 1).

**Figure 4.**
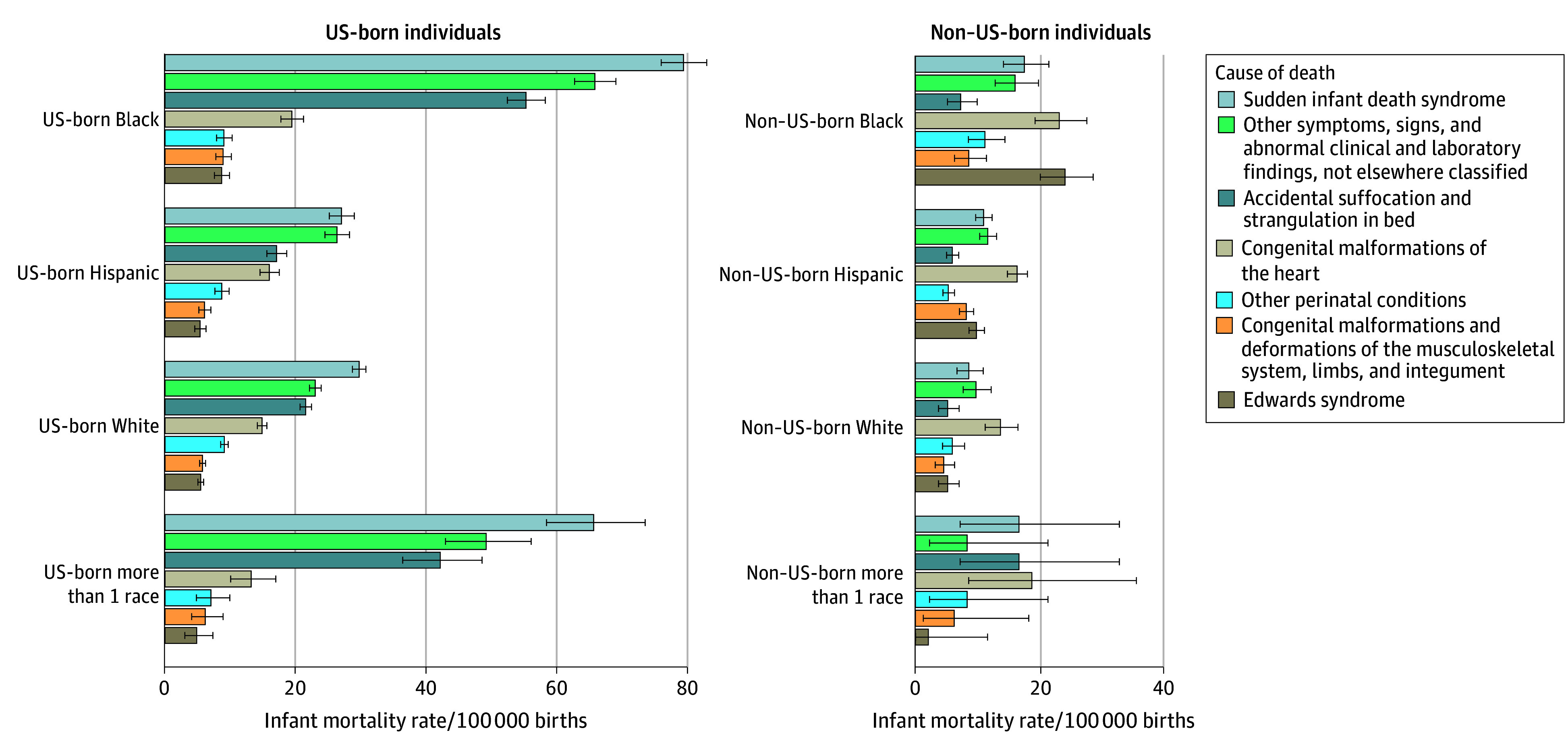
Infant Mortality Rate Among Full-Term Births by Cause of Death and Maternal Race Among US-Born Individuals and Non–US-Born Individuals Error bars denote 95% CIs.

The leading causes of death among full-term births included SIDS (31.7 deaths per 100 000 births), unspecified causes (26.4 deaths per 100 000 births), accidental suffocation and strangulation in bed (21.7 deaths per 100 000 births), congenital malformations of the heart (15.5 deaths per 100 000 births), other perinatal conditions (8.3 deaths per 100 000 births), Edwards syndrome (6.6 deaths per 100 000 births), and congenital malformations and deformations of the musculoskeletal system, limbs, and integument (6.3 deaths per 100 000 births). Among full-term births, rates of SIDS, unspecified causes, and accidental suffocation and strangulation in bed were higher among US-born vs non–US-born individuals (eFigure 3 in Supplement 1). Among US-born individuals, the distribution of leading causes of infant death in the full-term birth cohort was consistent across racial groups, with SIDS as the most frequent cause, followed by unspecified causes, and accidental suffocation and strangulation in bed. Among non–US-born individuals, the leading cause of death varied by race. Among Hispanic individuals, White individuals, and those identifying as more than 1 race, congenital malformations of the heart surpassed SIDS as the most common cause. Among Black, non–US-born individuals, Edwards syndrome was the leading cause, followed by congenital malformations of the heart (Figure 4).

### SUID Among Full-Term Births

US-born individuals had higher adjusted odds of SUID compared with non–US-born individuals (aOR, 2.90; 95% CI, 2.74-3.07). Higher adjusted odds of SUID were observed among US-born individuals identifying as Asian, Black, Hispanic, White, and more than 1 race (eTable 3 in Supplement 1). Across all study years, the crude SUID rate remained consistently higher among US-born individuals compared with their non–US-born counterparts (eFigure 4 in Supplement 1).

### Congenital Malformations, Deformations, and Chromosomal Abnormalities Among Full-Term Births

US-born individuals also had higher adjusted odds of infant death due to congenital malformations, deformations, and chromosomal abnormalities compared with non–US-born individuals (aOR, 1.07; 95% CI, 1.02-1.12). When stratified by race, higher adjusted odds were only observed among US-born White individuals. In contrast, among Black (models 1 and 2) and Hispanic (model 2) individuals, non–US-born individuals had higher odds of infant death from these causes compared with their US-born counterparts (eTable 4 in Supplement 1). Crude mortality rates for these causes were comparable between maternal nativity groups and declined over time. Rates were slightly higher among infants born to non–US-born individuals compared with US-born individuals (eFigure 5 in Supplement 1).

## Discussion

This population-based, retrospective, cohort study examined the association between maternal nativity status and infant mortality across multiple racial and ethnic groups stratified by gestational age. Our findings demonstrate that US-born individuals consistently have higher odds of infant mortality compared with their non–US-born counterparts and that these disparities are primarily seen in full-term births. We highlight that infant mortality varies not only by maternal race and ethnicity, as has been documented, but also by maternal nativity. Furthermore, we present differences in the leading causes of infant death by nativity and race and ethnicity among full-term births and show that US-born individuals have higher odds of SUID compared with non–US-born individuals.

Our findings are consistent with previous studies that have explored the intersections of race, ethnicity, nativity, and infant mortality. One study examining infant mortality among US-born and foreign-born individuals identifying as Latinx found that the infant mortality rate among births to the US-born Black Latinx group exceeded that of the US-born White Latinx group.^20^ That study also found that the infant mortality rate of births to foreign-born Black and White Latinx individuals was similar.^20^ Although the study did not directly compare the Black US-born and Black foreign-born groups, it suggests that racial disparities in infant mortality may be more pronounced among US-born populations. Our study extends this work by directly comparing US-born and non–US-born individuals within racial groups, and we similarly found higher infant mortality rates among US-born individuals across both Black and White populations. Another study investigating infant mortality among preterm infants of Hispanic mothers found differences in infant mortality rate by country and region of origin, but no significant difference by nativity status.^21^ This aligns with our findings because although we observed nativity-related differences among Hispanic individuals in the overall and full-term cohorts, no significant differences were found among preterm births. A third study focused only on term births among non-Latina White, African American, and Mexican American individuals and found that the infant mortality rate among US-born mothers in each of these groups exceeded that of their foreign-born counterparts.^22^ The disparities were particularly large for deaths attributed to SIDS.^22^ This aligns with our results, where SIDS was a leading cause of death among US-born individuals, within full-term births. Similarly, a study using New York City data (1995-1998) reported higher infant mortality rates among US-born mothers compared with foreign-born mothers.^23^ Their adjusted analyses showed that infants of foreign-born mothers were less likely to die from SIDS and external causes but more likely to die from congenital anomalies.^23^ In our study, we observed that SIDS, unspecified causes, and accidental suffocation and strangulation in bed were more common among infants of US-born individuals, whereas congenital malformations of the heart was a leading cause of death among infants of non–US-born individuals. Finally, we found that non–US-born Native Hawaiian or Other Pacific Islander individuals had higher infant mortality rates than US-born Native Hawaiian or Other Pacific Islander individuals in our overall and full-term cohorts, which contrasts the immigrant paradox observed in the other racial and ethnic groups. One study examining the association of preterm birth with maternal nativity, ethnicity, and race also confirmed the immigrant paradox for nearly all subgroups, except among Native Hawaiian or Other Pacific Islander individuals, for whom non–US-born birthing people had higher rates of preterm birth compared with their US-born counterparts.^24^

Our results support previous studies investigating racial and nativity-related disparities in SUID. Prior research examining temporal trends has documented higher SUID mortality rates among infants from mothers identifying as American Indian and Alaska Native, Black, and Native Hawaiian or Other Pacific Islander, compared with those identifying as Asian and White,^25^ highlighting persistent racial inequities. Another study reported that infants born to US-born women had more than a 3-fold higher SUID rate compared with those born to foreign-born women, a disparity observed across Asian, Black, Hispanic and White populations.^26^ Consistent with these findings, our study also found elevated odds of SUID among US-born individuals identifying as Asian, Black, Hispanic, White, and more than 1 race. Possible explanations for these disparities include differences in infant sleep practices, tobacco exposure, and infant feeding behaviors.^27,28^ However, further research is needed to better understand the underlying mechanisms contributing to these observed patterns.

We also found differences in congenital anomalies by race, ethnicity, and nativity. A prior study examining major birth defects by maternal nativity within racial and ethnic groups found that among non-Hispanic White mothers, approximately one-half of the defects occurred less frequently in foreign-born than in US-born mothers.^29^ In contrast, non-Hispanic Black mothers showed higher adjusted prevalence ratios for Patau syndrome and Edwards syndrome among foreign-born mothers, whereas Hispanic mothers had elevated ratios for spina bifida, anotia or microtia, and Down syndrome.^29^ In our analysis, which grouped all congenital malformations, deformations, and chromosomal abnormalities into a composite outcome, we found higher odds of anomalies among US-born White individuals and lower odds among US-born Black and Hispanic individuals (compared with their non–US-born counterparts), a pattern broadly consistent with these prior observations. These differences likely reflect a complex interplay of factors and the absence of a consistent trend between US-born and non–US-born individuals suggests that multiple protective and risk factors may be operating simultaneously.

### Limitations

Several limitations should be considered when interpreting our findings. First, birth records lack information on the duration of US residence for non–US-born birthing individuals, which may influence access to care and perinatal outcomes. Second, disaggregation into preterm and full-term cohorts resulted in smaller sample sizes for certain racial groups, such as American Indian and Alaska Native, which may explain the wider 95% CIs observed for these groups. Third, measures of poverty or environmental risk were not available despite being important determinants of infant mortality.^30,31^

## Conclusions

US-born individuals have higher odds of infant mortality compared with non–US-born individuals, and these disparities were primarily observed among full-term births. Race, ethnicity, and nativity status independently and jointly contribute to differences in infant mortality, with the most pronounced disparities observed among full-term births to US-born birthing individuals identifying as Black, Hispanic, White, or more than 1 race. Leading causes of infant death among full-term births vary by nativity, race, and ethnicity, with significantly higher rates of SUID among US-born individuals. These results highlight the impact of nativity, race, and ethnicity on infant mortality, emphasizing the need for investigation into the underlying factors contributing to these disparities among full-term births.

## Notes

Supplement 1. eTable 1. Cause of death classifications and corresponding *International Statistics Classification of Diseases and Related Health Problems, Tenth Revision (ICD-10)* codes eFigure 1. Infant mortality rate over time in the US by maternal nativity among all births, preterm births, and full-term births, 2016-2022 eTable 2. Frequency and rate of infant mortality by nativity and race in the US, 2016-2022 eFigure 2. Infant mortality rate among preterm births by cause of death among US-born individuals and non–US-born individuals eFigure 3. Infant mortality rate among full-term births by cause of death among US-born individuals and non–US-born individuals eTable 3. Frequency and rate of sudden unexpected infant death (SUID) among full-term births by nativity and race in the US eFigure 4. Infant mortality rate by sudden unexpected infant death (SUID) among full-term births over time in the US by maternal nativity, 2016-2022 eTable 4. Frequency and rate of deaths due to congenital malformations, deformations, and chromosomal abnormalities among full-term births by nativity and race in the US eFigure 5. Infant mortality rate by congenital malformations, deformations, and chromosomal abnormalities (Q00-Q99) among full-term births over time in the US by maternal nativity, 2016-2022

Supplement 2. Data Sharing Statement
