# Supplementary material for: Maternal Nativity, Race, and Ethnicity and Infant Mortality in the US
Source: JAMA Netw Open. 2026 Jan 6;9(1):e2552230. doi: 10.1001/jamanetworkopen.2025.52230 (PMC12776206; doi:10.1001/jamanetworkopen.2025.52230)
Supplement: Supplement 1. — eTable 1. Cause of death classifications and corresponding International Statistics Classification of Diseases and Related Health Problems, Tenth Revision (ICD-10) codes eFigure 1. Infant mortality rate over time in the US by maternal nativity among all births, preterm births, and full-term births, 2016-2022 eTable 2. Frequency and rate of infant mortality by nativity and race in the US, 2016-2022 eFigure 2. Infant mortality rate among preterm births by cause of death among US-born individuals and non–US-born individuals eFigure 3. Infant mortality rate among full-term births by cause of death among US-born individuals and non–US-born individuals eTable 3. Frequency and rate of sudden unexpected infant death (SUID) among full-term births by nativity and race in the US eFigure 4. Infant mortality rate by sudden unexpected infant death (SUID) among full-term births over time in the US by maternal nativity, 2016-2022 eTable 4. Frequency and rate of deaths due to congenital malformations, deformations, and chromosomal abnormalities among full-term births by nativity and race in the US eFigure 5. Infant mortality rate by congenital malformations, deformations, and chromosomal abnormalities (Q00-Q99) among full-term births over time in the US by maternal nativity, 2016-2022 [file jamanetwopen-e2552230-s001.pdf]

Supplemental Online Content

Christodoulakis N, Muraca GM. Maternal nativity, race, and ethnicity and infant mortality in the US. *JAMA Netw Open*. 2026;9(1):e2552230.
doi:10.1001/jamanetworkopen.2025.52230

eTable 1. Cause of death classifications and corresponding *International Statistics Classification of Diseases and Related Health Problems, Tenth Revision (ICD-10)* codes

eFigure 1. Infant mortality rate over time in the US by maternal nativity among all births, preterm births, and full-term births, 2016-2022

eTable 2. Frequency and rate of infant mortality by nativity and race in the US, 2016-2022

eFigure 2. Infant mortality rate among preterm births by cause of death among US-born individuals and non–US-born individuals

eFigure 3. Infant mortality rate among full-term births by cause of death among US-born individuals and non–US-born individuals

eTable 3. Frequency and rate of sudden unexpected infant death (SUID) among full-term births by nativity and race in the US

eFigure 4. Infant mortality rate by sudden unexpected infant death (SUID) among full-term births over time in the US by maternal nativity, 2016-2022

eTable 4. Frequency and rate of deaths due to congenital malformations, deformations, and chromosomal abnormalities among full-term births by nativity and race in the US

eFigure 5. Infant mortality rate by congenital malformations, deformations, and chromosomal abnormalities (Q00-Q99) among full-term births over time in the US by maternal nativity, 2016-2022

This supplemental material has been provided by the authors to give readers additional information about their work.

© 2026 Christodoulakis N et al. *JAMA Network Open*.

eTable 1. Cause of death classifications and corresponding *International Statistics Classification of Diseases and Related Health Problems, Tenth Revision (ICD-10)* codes

**Cause of Death**	**ICD-10 Code(s)**
Accidental suffocation and strangulation in bed	W75
Bacterial sepsis of newborn	P36
Congenital malformations and deformations of the musculoskeletal system, limbs, and integument	Q65-Q85
Congenital malformations of the heart	Q20-Q24
Edwards syndrome	Q91.0-Q91.3
Extremely low birthweight or extreme immaturity	P07.0, P07.2
Newborn affected by premature rupture of membranes	P01.1
Other low birthweight or preterm[a]	P07.1, P07.3
Other perinatal conditions[b]	P29, P70.3-P70.9, P71-P76, P78-P81, P83.0-P83.1, P83.3-P83.9, P90-P96
Other symptoms, signs, and abnormal clinical and laboratory findings, not elsewhere classified	R00-R53, R55-R94, R96-R99
Respiratory distress of newborn	P22
Sudden infant death syndrome	R95

[a] P07.1: birth weight 1000-2499 grams; P07.3: 28 completed weeks or more but less than 37 completed weeks (196 completed days but less than 259 completed days) of gestation.

[b] P29: cardiovascular disorders originating in the perinatal period; P70.3: iatrogenic neonatal hypoglycaemia; P70.4: other neonatal hypoglycaemia; P70.8: other transitory disorders of carbohydrate metabolism of fetus and newborn; P70.9: transitory disorder of carbohydrate metabolism of fetus and newborn, unspecified; P71: transitory neonatal disorders of calcium and magnesium metabolism; P72: other transitory neonatal endocrine disorders; P74: other transitory neonatal electrolyte and metabolic disturbances; P75: meconium ileus in cystic fibrosis; P76: other intestinal obstruction of newborn; P78: other perinatal digestive system disorders; P80: hypothermia of newborn; P81: other disturbances of temperature regulation of newborn; P83.0: sclerema neonatorum; P83.1: neonatal erythema toxicum; P83.3: other and unspecified oedema specific to fetus and newborn; P83.4: breast engorgement of newborn; P83.5: congenital hydrocele; P83.6: umbilical polyp of newborn; P83.8: other specified conditions of integument specific to fetus and newborn; P83.9: condition of integument specific to fetus and newborn, unspecified; P90: convulsions of newborn; P91: other disturbances of cerebral status of newborn; P92: feeding problems of newborn; P93: reactions and intoxications due to drugs administered to fetus and newborn; P94: disorders of muscle tone of newborn; P95: fetal death of unspecified cause; P96: other conditions originating in the perinatal period.

© 2026 Christodoulakis N et al. *JAMA Network Open*.

eFigure 1. Infant mortality rate over time in the US by maternal nativity among all births, preterm births, and full-term births, 2016-2022

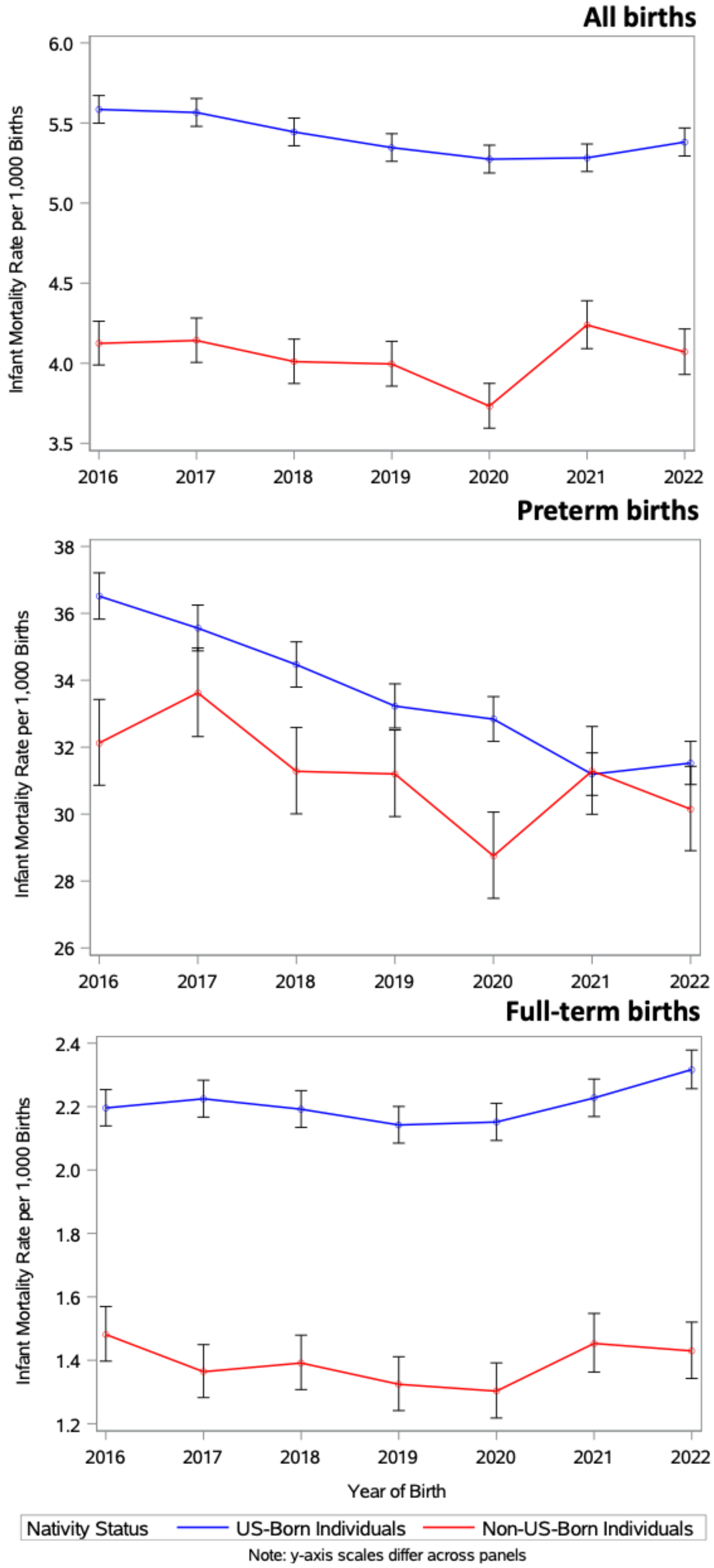

© 2026 Christodoulakis N et al. *JAMA Network Open*.

eTable 2. Frequency and rate of infant mortality by nativity and race in the US, 2016-2022

Maternal nativity and self-reported maternal race	Number of births	Number of infant deaths	Rate of infant death per 1,000 births	Crude odds ratio (95% CI)	Adjusted[a] odds ratio (95% CI)	Adjusted[b] odds ratio (95% CI)
**All births (n = 24,904,524)**						
US-born AIAN	184,704	1,463	7.9	1.78 (0.96-3.32)	1.46 (0.78-2.73)	1.42 (0.74-2.72)
Non-US-born AIAN	2,242	10	4.5	Reference	Reference	Reference
US-born Asian	317,344	1,130	3.6	1.14 (1.06-1.21)	1.12 (1.05-1.20)	1.04 (0.96-1.12)
Non-US-born Asian	1,241,049	3,894	3.1	Reference	Reference	Reference
US-born Black	2,983,124	31,449	10.5	1.59 (1.54-1.65)	1.48 (1.42-1.53)	1.14 (1.09-1.18)
Non-US-born Black	593,756	3,945	6.6	Reference	Reference	Reference
US-born Hispanic	3,220,173	15,500	4.8	1.15 (1.12-1.18)	1.27 (1.24-1.30)	1.14 (1.10-1.17)
Non-US-born Hispanic	2,752,646	11,530	4.2	Reference	Reference	Reference
US-born NHOPI	22,392	159	7.1	0.92 (0.76-1.12)	1.03 (0.84-1.27)	0.98 (0.78-1.23)
Non-US-born NHOPI	38,035	292	7.7	Reference	Reference	Reference
US-born White	12,137,978	51,928	4.3	1.46 (1.40-1.52)	1.30 (1.25-1.35)	1.16 (1.11-1.21)
Non-US-born White	848,827	2,499	2.9	Reference	Reference	Reference
US-born more than one race	509,883	3,258	6.4	1.62 (1.41-1.87)	1.32 (1.14-1.52)	1.19 (1.02-1.39)
Non-US-born more than one race	52,371	207	4.0	Reference	Reference	Reference
**Preterm births (n = 2,469,939)**						
US-born AIAN	21,116	780	36.9	1.12 (0.52-2.38)	0.98 (0.46-2.10)	1.01 (0.43-2.37)
Non-US-born AIAN	211	7	33.2	Reference	Reference	Reference
US-born Asian	30,351	801	26.4	1.00 (0.93-1.09)	1.00 (0.92-1.09)	1.01 (0.92-1.12)
Non-US-born Asian	104,122	2,741	26.3	Reference	Reference	Reference
US-born Black	440,477	21,773	49.4	1.04 (1.00-1.08)	0.97 (0.93-1.01)	0.94 (0.89-0.98)
Non-US-born Black	59,780	2,850	47.7	Reference	Reference	Reference
US-born Hispanic	322,213	10,140	31.5	1.03 (1.00-1.06)	1.03 (1.00-1.07)	1.02 (0.98-1.06)
Non-US-born Hispanic	256,220	7,863	30.7	Reference	Reference	Reference
US-born NHOPI	2,380	110	46.2	1.36 (1.06-1.75)	1.32 (1.01-1.73)	1.24 (0.91-1.70)
Non-US-born NHOPI	4,482	154	34.4	Reference	Reference	Reference
US-born White	1,107,723	30,964	28.0	1.08 (1.03-1.14)	1.00 (0.95-1.05)	0.99 (0.93-1.05)
Non-US-born White	62,359	1,611	25.8	Reference	Reference	Reference
US-born more than one race	53,992	1,927	35.7	1.22 (1.02-1.46)	1.07 (0.89-1.29)	1.08 (0.88-1.34)
Non-US-born more than one race	4,513	133	29.5	Reference	Reference	Reference

© 2026 Christodoulakis N et al. *JAMA Network Open*.

**Full-term births (n = 22,434,585)**						
US-born AIAN	163,588	683	4.2	2.83 (0.91-8.79)	2.14 (0.69-6.67)	2.11 (0.68-6.58)
Non-US-born AIAN	2,031	3	1.5	Reference	Reference	Reference
US-born Asian	286,993	329	1.1	1.13 (1.00-1.28)	1.12 (0.98-1.27)	1.13 (0.99-1.28)
Non-US-born Asian	1,136,927	1,153	1.0	Reference	Reference	Reference
US-born Black	2,542,647	9,676	3.8	1.86 (1.75-1.98)	1.55 (1.45-1.66)	1.54 (1.43-1.65)
Non-US-born Black	533,976	1,095	2.1	Reference	Reference	Reference
US-born Hispanic	2,897,960	5,360	1.8	1.26 (1.21-1.31)	1.39 (1.32-1.45)	1.34 (1.27-1.40)
Non-US-born Hispanic	2,496,426	3,667	1.5	Reference	Reference	Reference
US-born NHOPI	20,012	49	2.4	0.59 (0.43-0.82)	0.73 (0.52-1.03)	0.75 (0.53-1.07)
Non-US-born NHOPI	33,553	138	4.1	Reference	Reference	Reference
US-born White	11,030,255	20,964	1.9	1.69 (1.58-1.80)	1.45 (1.35-1.55)	1.39 (1.30-1.49)
Non-US-born White	786,468	888	1.1	Reference	Reference	Reference
US-born more than one race	455,891	1,331	2.9	1.89 (1.50-2.39)	1.33 (1.04-1.69)	1.31 (1.02-1.67)
Non-US-born more than one race	47,858	74	1.5	Reference	Reference	Reference

Abbreviations: AIAN, American Indian or Alaska Native; NHOPI, Native Hawaiian and Other Pacific Islander.

[a] Model 1: adjusted for maternal age, maternal education, maternal marital status, maternal insurance status, maternal cigarette use, and prenatal care.

[b] Model 2: adjusted for maternal age, maternal education, maternal marital status, maternal insurance status, maternal cigarette use, prenatal care, infant birth weight, severe neonatal morbidity, and neonatal intensive care unit admission.

© 2026 Christodoulakis N et al. *JAMA Network Open*.

eFigure 2. Infant mortality rate among preterm births by cause of death among US-born individuals and non–US-born individuals

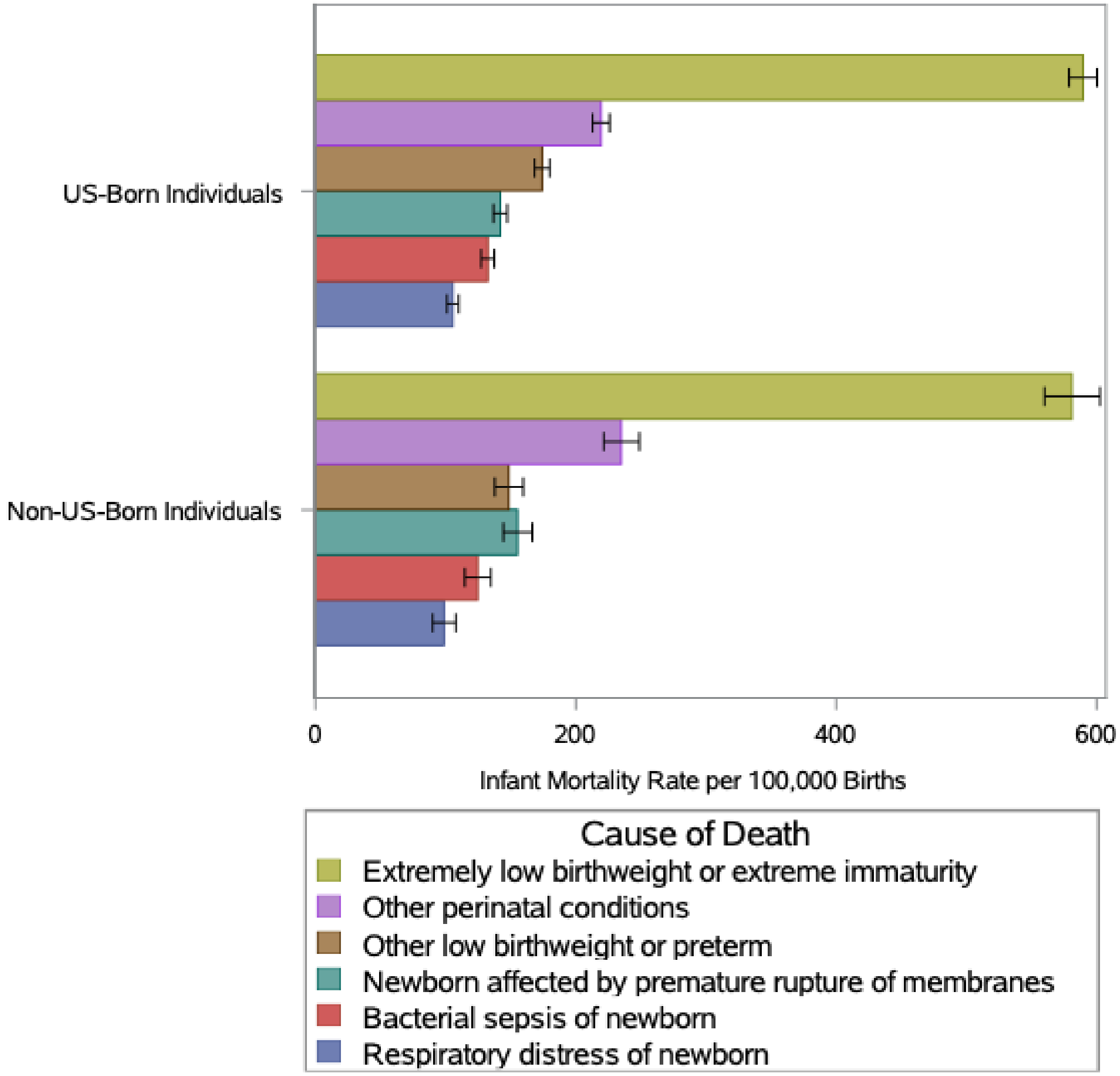

© 2026 Christodoulakis N et al. *JAMA Network Open*.

eFigure 3. Infant mortality rate among full-term births by cause of death among US-born individuals and non–US-born individuals

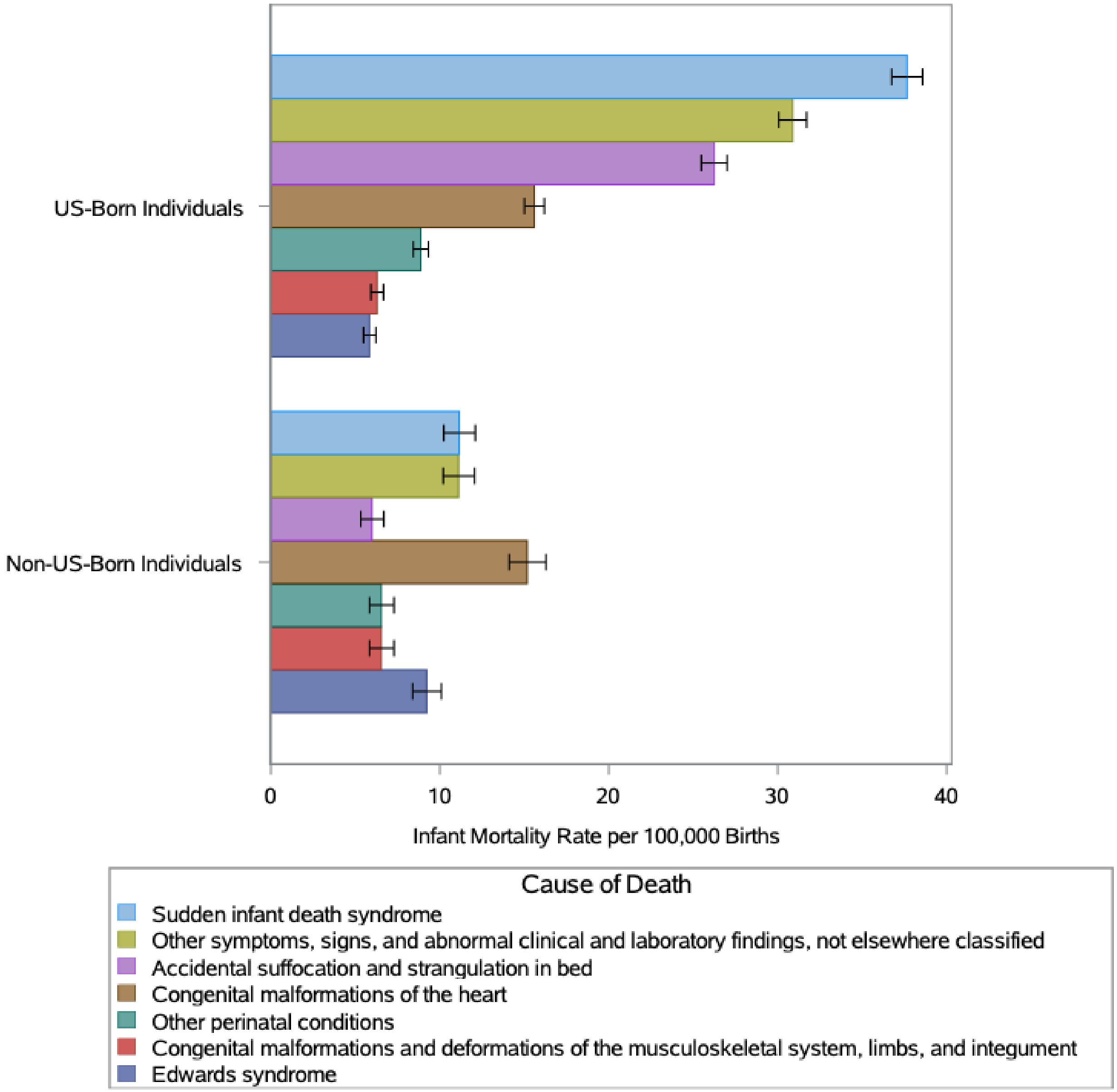

© 2026 Christodoulakis N et al. *JAMA Network Open*.

eTable 3. Frequency and rate of sudden unexpected infant death (SUID) among full-term births by nativity and race in the US

Maternal nativity and self-reported maternal race	Number of infant deaths	Rate of infant death per 100,000 births	Crude odds ratio (95% CI)	Adjusted[a] odds ratio (95% CI)	Adjusted[b] odds ratio (95% CI)
**Full-term births (n = 22,434,585)**					
US-born AIAN	323	197.4	4.02 (0.56-28.61)	2.80 (0.39-19.98)	2.80 (0.39-20.00)
Non-US-born AIAN	1	49.2	Reference	Reference	Reference
US-born Asian	100	34.8	1.82 (1.43-2.30)	1.65 (1.28-2.13)	1.65 (1.28-2.12)
Non-US-born Asian	218	19.2	Reference	Reference	Reference
US-born Black	5,081	199.8	4.90 (4.28-5.61)	3.30 (2.87-3.81)	3.23 (2.80-3.72)
Non-US-born Black	218	40.8	Reference	Reference	Reference
US-born Hispanic	2,021	69.7	2.47 (2.26-2.69)	2.38 (2.17-2.62)	2.37 (2.16-2.61)
Non-US-born Hispanic	706	28.3	Reference	Reference	Reference
US-born NHOPI	28	139.9	0.84 (0.53-1.32)	0.87 (0.54-1.42)	0.87 (0.54-1.41)
Non-US-born NHOPI	56	166.9	Reference	Reference	Reference
US-born White	8,106	73.5	3.14 (2.72-3.64)	2.12 (1.83-2.45)	2.12 (1.83-2.46)
Non-US-born White	184	23.4	Reference	Reference	Reference
US-born more than one race	711	156.0	3.93 (2.49-6.20)	2.34 (1.47-3.73)	2.34 (1.47-3.73)
Non-US-born more than one race	19	39.7	Reference	Reference	Reference

Abbreviations: AIAN, American Indian or Alaska Native; NHOPI, Native Hawaiian and Other Pacific Islander.
[a] Model 1: adjusted for maternal age, maternal education, maternal marital status, maternal insurance status, maternal cigarette use, and prenatal care.
[b] Model 2: adjusted for maternal age, maternal education, maternal marital status, maternal insurance status, maternal cigarette use, prenatal care, infant birth weight, severe neonatal morbidity, and neonatal intensive care unit admission.

© 2026 Christodoulakis N et al. *JAMA Network Open*.

eFigure 4. Infant mortality rate by sudden unexpected infant death (SUID) among full-term births over time in the US by maternal nativity, 2016-2022

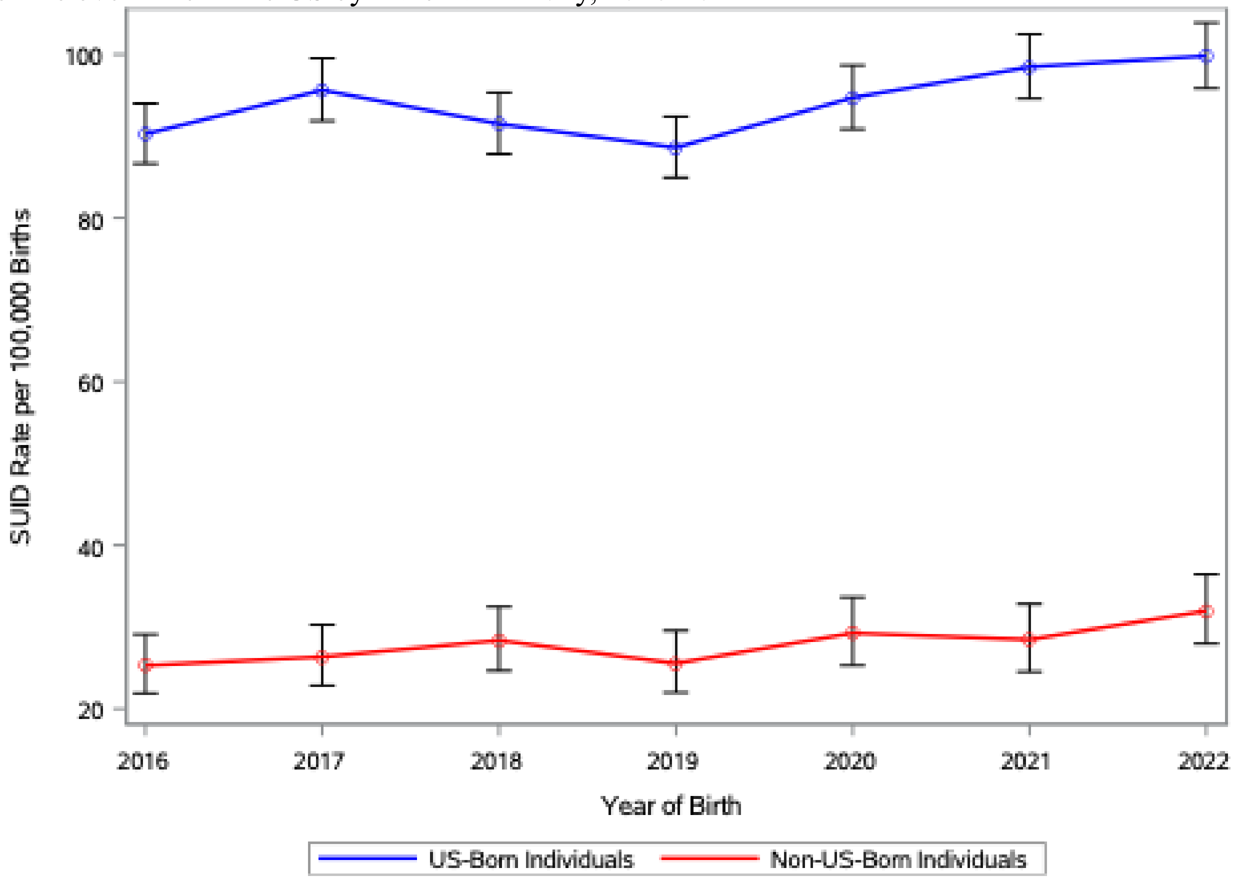

© 2026 Christodoulakis N et al. *JAMA Network Open*.

eTable 4. Frequency and rate of deaths due to congenital malformations, deformations, and chromosomal abnormalities among full-term births by nativity and race in the US

**Maternal nativity and self-reported maternal race**	**Number of infant deaths**	**Rate of infant death per 100,000 births**	**Crude odds ratio (95% CI)**	**Adjusted[a] odds ratio (95% CI)**	**Adjusted[b] odds ratio (95% CI)**
**Full-term births (n = 22,434,585)**					
US-born AIAN	123	75.2	1.52 (0.21-10.87)	1.45 (0.20-10.41)	1.30 (0.18-9.45)
Non-US-born AIAN	1	49.2	Reference	Reference	Reference
US-born Asian	87	30.3	0.88 (0.70-1.11)	0.95 (0.74-1.21)	0.97 (0.76-1.24)
Non-US-born Asian	391	34.4	Reference	Reference	Reference
US-born Black	1,686	66.3	0.73 (0.66-0.80)	0.87 (0.78-0.98)	0.85 (0.76-0.95)
Non-US-born Black	488	91.4	Reference	Reference	Reference
US-born Hispanic	1,525	52.6	0.78 (0.72-0.83)	0.98 (0.90-1.06)	0.90 (0.83-0.98)
Non-US-born Hispanic	1,692	67.8	Reference	Reference	Reference
US-born NHOPI	9	45.0	0.58 (0.27-1.24)	0.79 (0.36-1.75)	0.86 (0.39-1.91)
Non-US-born NHOPI	26	77.5	Reference	Reference	Reference
US-born White	5,446	49.4	1.08 (0.97-1.20)	1.16 (1.04-1.29)	1.09 (0.97-1.21)
Non-US-born White	361	45.9	Reference	Reference	Reference
US-born more than one race	221	48.5	1.29 (0.80-2.08)	1.39 (0.84-2.29)	1.37 (0.83-2.27)
Non-US-born more than one race	18	37.6	Reference	Reference	Reference

Abbreviations: AIAN, American Indian or Alaska Native; NHOPI, Native Hawaiian and Other Pacific Islander.

[a] Model 1: adjusted for maternal age, maternal education, maternal marital status, maternal insurance status, maternal cigarette use, and prenatal care.

[b] Model 2: adjusted for maternal age, maternal education, maternal marital status, maternal insurance status, maternal cigarette use, prenatal care, infant birth weight, severe neonatal morbidity, and neonatal intensive care unit admission.

© 2026 Christodoulakis N et al. *JAMA Network Open*.

eFigure 5. Infant mortality rate by congenital malformations, deformations, and chromosomal abnormalities (Q00-Q99) among full-term births over time in the US by maternal nativity, 2016-2022

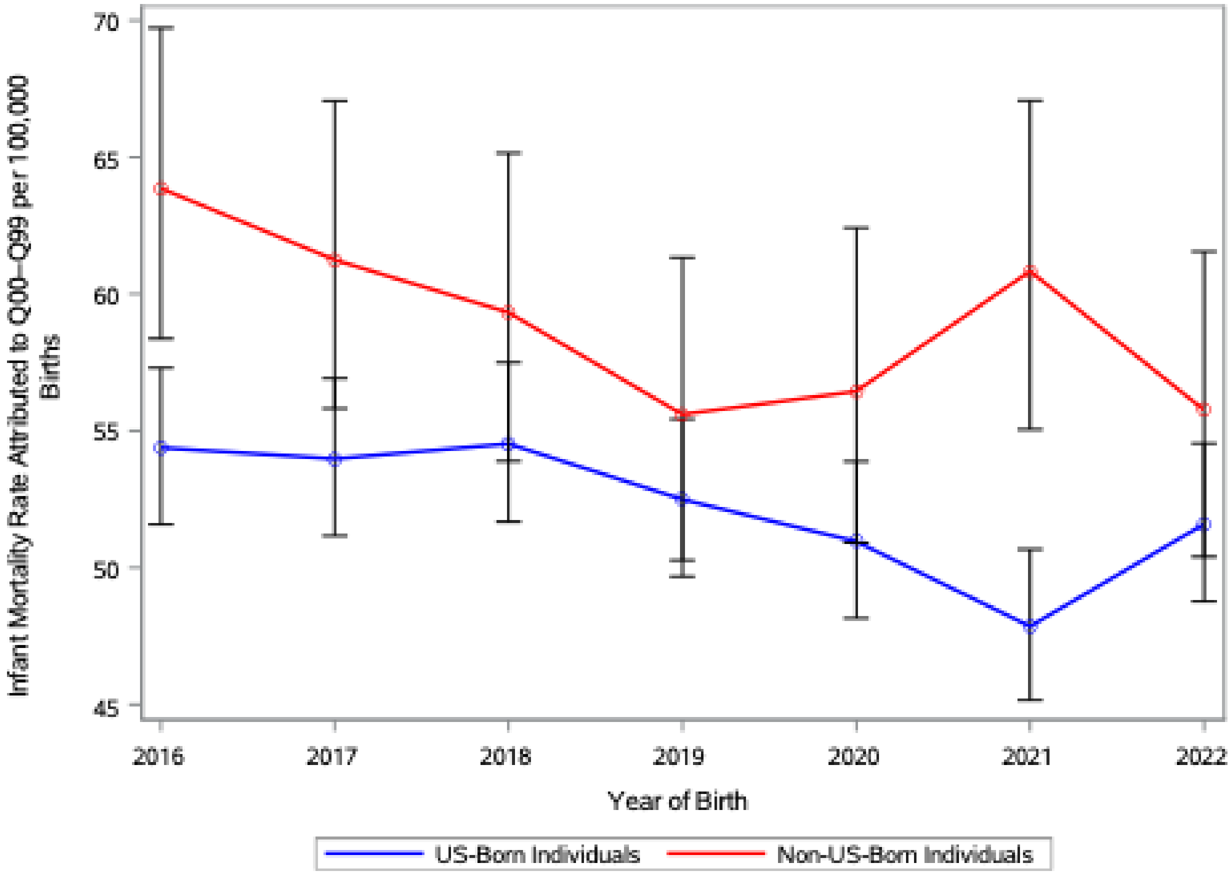

© 2026 Christodoulakis N et al. *JAMA Network Open*.
