# Supplementary material for: Maternal Nativity, Race, and Ethnicity and Infant Mortality in the US
Source: JAMA Netw Open. 2026 Jan 6;9(1):e2552230. doi: 10.1001/jamanetworkopen.2025.52230 (PMC12776206; doi:10.1001/jamanetworkopen.2025.52230)
Supplement: Supplement 2. — Data Sharing Statement [file jamanetwopen-e2552230-s002.pdf]

Data Sharing Statement

Christodoulakis. Maternal Nativity, Race, and Ethnicity and Infant Mortality in the US. *JAMA Netw Open*. Published January 06, 2026. doi:10.1001/jamanetworkopen.2025.52230

Data
Data available: Yes
Data types: Deidentified participant data, Data dictionary
How to access data: The data from this study can be obtained from the National Vital Statistics System (NVSS) Period/Cohort Linked Birth-Infant Death Data Files: https://www.cdc.gov/nchs/data_access/vitalstatsonline.htm
When available: With publication

Supporting Documents
Document types: None

Additional Information
Who can access the data: Anyone requesting the data
Types of analyses: For any purpose
Mechanisms of data availability: Without investigator support
